# Rapid saccadic response with fearful gaze cue

**DOI:** 10.1371/journal.pone.0212450

**Published:** 2019-03-07

**Authors:** Reiko Matsunaka, Kazuo Hiraki

**Affiliations:** Graduate School of Arts & Sciences, The University of Tokyo, Tokyo, Japan; Harvard Medical School, UNITED STATES

## Abstract

It has been shown that an averted gaze with emotional expression guides our attention toward a gazed-at location, and the effect of a gaze with fearful expression has been well-investigated. However, the findings are not consistent, and most studies used the manual response measure. Recent studies suggest that examining eye movements is more suitable to capture the early stage of the effect of threat-related stimuli on attentional process. Therefore, in the present study, we investigated the effects of static neutral and fearful gaze on overt attention orienting by examining the saccadic responses in an unselected sample of people. Our results found the gaze congruency effects for both expressions, and importantly, enhanced attention orienting by fearful gaze at a short stimulus onset asynchrony (SOA): participants looked faster at the fearful gaze-cued target than the neutral gaze-cued one at the 300 ms SOA. These findings provide the first evidence that fearful averted gaze elicits rapid overt attention orienting toward the target, and suggest that the information of gaze direction and emotional expression are rapidly integrated and modulate the oculomotor system.

## Introduction

Attention orienting is twofold: one is covert orienting that is achieved without head and/or eye movements; the other is overt orienting that is accompanied by head and/or eye movements. Overt orienting toward an object improves the efficiency of object processing [1] and focusing on overt orienting reveals the allocation of attention in real time [2]. We often orient toward an object guided by social signals, such as others’ gaze directions, facial expressions, gestures, verbal instructions, and so on. Previous research demonstrated that gaze direction reflexively triggers our attention toward the gaze-cued side, even when we try to ignore it [3]. In a standard gaze-cueing paradigm, a schematic or photographic neutral face with averted gaze is presented centrally as a cue, and participants are required to respond to the target that appears on either the congruent or incongruent side of the gaze direction. Results showed that participants’ manual reaction times (RTs) or saccadic reaction times (SRTs) were shorter when the target appeared on the congruent side than when it appeared on the incongruent side [3–6]. This is called the gaze congruency effect (GCE) and demonstrates that gaze direction influences our covert and overt attention orienting.

Furthermore, previous studies have investigated how gaze direction with emotional expression influences our covert attention orienting; particularly, the effect of fearful gaze has been well-investigated. The findings demonstrated that the magnitude of the GCE (the RT difference between congruent and incongruent conditions) was robustly larger with fearful gaze than with neutral gaze among high anxiety individuals [7–9]. On the other hand, the findings in the non-anxious or general population are not consistent [10–17]. In fact, there are many methodological differences among previous research; therefore, it is difficult to compare the results directly, and how emotional gaze influences our covert attention orienting is still controversial. For example, Hietanen & Leppänen (2003) [12] have conducted a series of experiments and demonstrated that the effects of gaze direction and facial expressions on RTs are independent: the robust GCE was found, but the magnitudes were not different among facial expressions (e.g. neutral, happy, angry, or fearful). Alternatively, Graham et al. (2010) [11] showed that morphed neutral to emotional gaze (e.g. fearful, disgusted, or happy gaze) shortened the RTs relative to neutral gaze, and the magnitude was larger with emotional gaze than with neutral gaze at long stimulus-onset-asynchrony (SOA) condition (e.g. 1000 ms). Similarly, it is also reported that the magnitude with fearful gaze was larger than with happy, angry, or neutral gaze among non-anxious participants [10, 13–17]

One possible explanation for the inconsistent results with fearful gaze is the less naturalistic experimental situation. Since the face stimuli were presented via display and as static and/or schematic cues, they might not act as a signal for potential danger and led to null findings (e.g. [12]). Previous research, which used a dynamic cue sequence, often reported the emotional modulation of the GCEs [9, 11, 14, 18], and it might support this view. Another possible factor is the response mode used in the previous studies. When observing a person who is looking at something with a fearful expression, the natural response may be to check for a potentially dangerous source by looking at it; however, to the best of our knowledge, most of the previous studies investigating neutral and/or emotional gaze-triggered attention orienting used the manual response mode to measure participant’s behavioral response, and the effects on eye movement were not investigated. Therefore, it has been shown that gaze direction with neutral expression triggers our rapid overt attention orienting [5, 6]; however, it remains unclear how gaze direction with fearful expression influences our overt attention orienting. Recent research has shown that examining eye movement behaviours instead of manual response provide more direct insight into the temporal and spatial dynamics of the effects of threat-related emotion on attention [19–24]. Thus, using the saccade response mode and focusing on our overt attention would be more suitable to examine the gaze-triggered attention orienting with fearful gaze.

In the present study, we investigate the effect of neutral and fearful gaze on overt attention orienting by examining saccadic response with an eye-tracker. It has been reported that the neutral gaze that facilitated the saccadic response toward the target appeared on the congruent side [5, 6], and the fearful gaze also produced the robust GCE in the manual response mode. Therefore, we expected that both neutral and fearful gaze would induce the GCE: the SRTs toward the target that appeared on the congruent side are shorter than those for the target on the incongruent side. Furthermore, it is reported that fearful gaze induced more rapidly saccadic response relative to neutral gaze in 12-month-old infants [25] and a magnetoencephalography (MEG) research showed that fearful gaze elicited a very early attention influence on gaze-cued target processing in adults [26]. Thus, we expected that fearful gaze would induce more rapid saccadic response toward the gaze-cued target compared with the neutral gaze: the SRTs are shorter with fearful averted gaze than with neutral averted gaze when the target appeared on the congruent side.

## Methods

### Participants

Eighteen adults (10 male and eight female; age: M = 23.8 years, SD = 4.8 years) participated in the present study. The sample size was similar to previous gaze-triggered overt attention orienting studies [5, 6, 27] and to several studies on effects of threat-related stimuli on saccadic response (e.g. [19, 20, 28]). All participants were healthy and reported normal or corrected-to-normal vision, and participants were not screened for anxiety or autism. Four additional participants were excluded because they did not provide appropriate calibration data, which included large error vectors and/or missing data in the calibrated points. All participants provided written informed consent. The ethics committee of The University of Tokyo approved the experiments.

### Apparatus

Participants were seated and faced the 23-inch wide colour monitor (screen resolution 1920 × 1080 pixels) of an infrared bright pupil eye-tracking system (300Hz, Tobii TX300, Tobii Technology AB) placed on a table. The accuracy of the eye-tracker was 0.5° on average for binocular vision. The monitor was about 65 cm away from the participant, and curtains surrounded the table. One camera was set on the table, another camera was set behind the participant, and the two images were synchronized with a quad splitter (YH-446C, Mother Tool) to monitor participant behaviours. The stimulus presentations were controlled by E-Prime 2.0 software (Psychology Software Tools).

### Stimuli

The face stimuli were grayscale images of two female models portraying neutral or fearful expressions from the ATR Facial Expression Image database (DB99, ATR-Promotions), which were used in Matsunaka & Hiraki (2014) [25]. The direction of eye gaze on these images was manipulated with Adobe Photoshop to create stimuli with leftward and rightward gaze directions. The face pictures were 7.5° in height and 5.1° in width, and the eye region subtended about 1.2° in height and 3.2° in width. Peripheral targets were black-and-white 2 by 4 checkerboard pattern on rectangular objects subtending a visual angle of 2.9° in height and 1.7° in width and presented 8.8° to the left or right of the centre of the screen.

### Design and procedure

**Fig 1:**
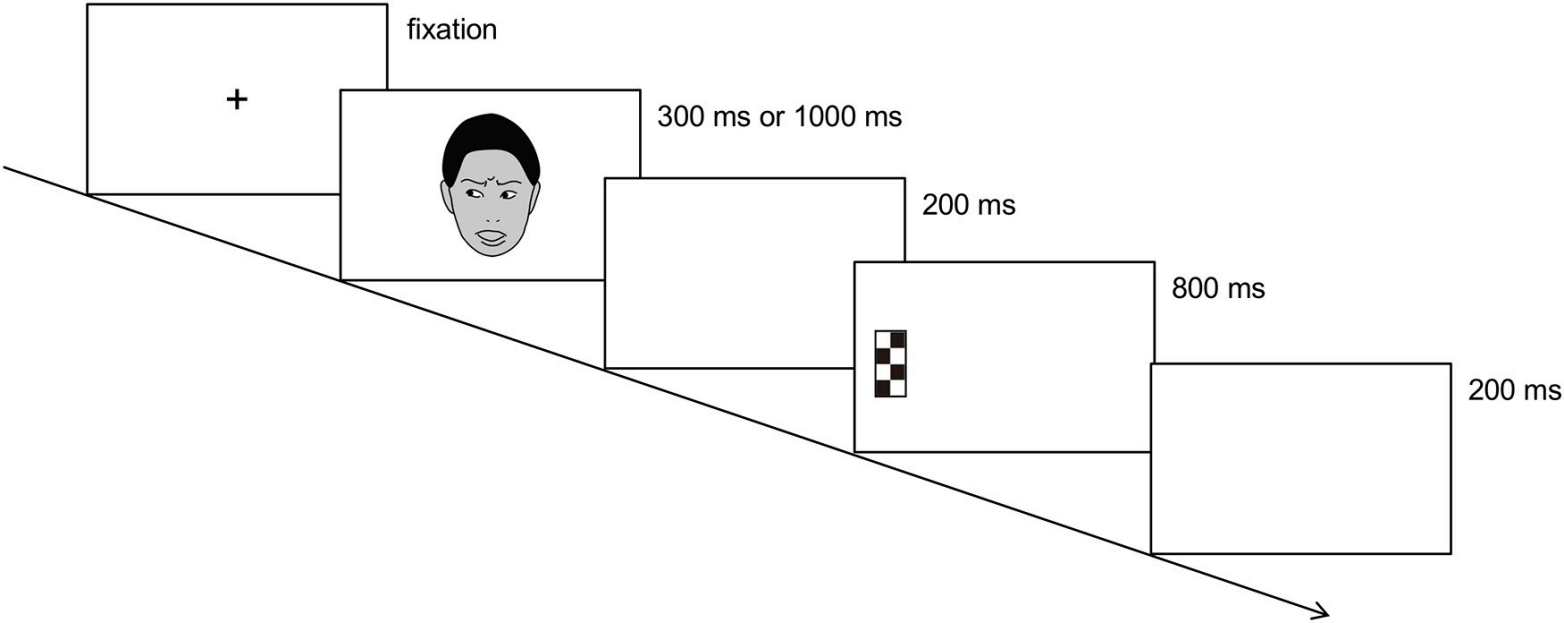
Example of a stimulus presentation sequence. The expression is fearful; the target appears on the incongruent side. At the beginning of each trial, a fixation cross was presented on the monitor. Then a static face with a leftward, rightward or forward gaze direction showing a neutral or fearful expression was presented for 300 ms or 1000 ms. The monitor then displayed a blank screen for 200 ms, followed by the presentation of a leftward or rightward peripheral target for 800 ms. Another blank screen was then presented for 200 ms, and the next trial started. The images of faces shown here do not depict the actual stimuli but are intended only as examples.

Participants’ eye movements were recorded with the Tobii Studio 3.2.1 software, and a standard five-point (the upper and lower left and right corners and the centre) calibration procedure was performed until more than three calibration points in each eye were successful in the software [29]. The experiment began after successful calibration. At the beginning of each trial, a fixation point was presented. After 1000 ms, a face with leftward, rightward or forward gaze direction showing a neutral or fearful expression was presented for 300 ms or 1000 ms (Fig 1). SOAs of 300 ms and 1000 ms were selected because previous studies investigating the effect of neutral gaze on the saccadic response used the same SOA [5, 6]; in addition, an SOA of 1000 ms was selected because the effects of fearful gaze on saccadic response were found with this SOA in previous infant research [25] and because previous adult studies reported the effect of fearful gaze on the manual response were found at long, rather than short SOAs [11]. Then, a peripheral target appeared either to the left or right side of the face. Both a face and a target remained on the screen for 800 ms. The monitor then showed a blank screen for 200 ms, and the next trial started. All stimuli were presented on a uniform white background. Participants were instructed to first fixate upon the fixation point and to make saccades to the target when it appeared. We emphasized that the gaze direction or the facial expression did not predict the side on which the target would appear.

The experimental design used three within-subject factors: SOA (300 ms and 1000 ms), facial expression (neutral and fearful), and congruency (congruent, incongruent, and direct gaze). The target appeared equally often to the right or left side; face identity, gaze direction, and emotional expression were independently varied. The experiment consisted of 18 practice trials followed by 576 test trials presented in 12 blocks (six blocks with SOA 300 ms condition; six blocks with SOA 1000 ms condition) of 48 trials. The order of the duration of SOA was counterbalanced across participants. Calibration and drift correction of the position signal were repeated every three blocks to ensure accuracy during the experiment.

### Data analysis

On each trial, a graph of recorded horizontal gaze position and velocity data versus time was generated during target presentation, and saccades were identified manually if velocity exceeded 30 °/s. Similarly, the end of a saccade was defined as a gaze position when it fell within an area of 0.5° for 10 consecutive points of gaze data (about 35 ms). After a saccade was determined, SRT was calculated by subtracting the target onset time from the saccade onset time. A trial was excluded if the SRT was below 80 ms as an anticipatory saccade [30]; if the SRT was three standard deviations above the overall mean value; if there were any missing data points due to movement or blinking during the defined saccade; if the total face-orientation time was less than half of the face cue duration; or if there were eye movements in the wrong direction.

The number of excluded trials and SRTs were analysed with a repeated-measures analysis of variance (ANOVA), with SOA (300 ms and 1000 ms), facial expression (neutral and fearful), and gaze congruency (congruent, incongruent and direct gaze) as within-subject factors; and if significant main effects or interaction were found, the Holm’s sequentially rejective Bonferroni procedure was used as a post hoc test. The magnitude of GCE was analysed with the planned paired t-test.

## Results

The data is available on Open Science Framework database (https://osf.io/cpyda/).

### Excluded trials

Trials were rejected for anticipatory eye movements (SRT below 80 ms; 0.5%), for exceeding the three standard deviation criteria (1.2%), for blinks or poor validity (9.8%), for the less than half of the face cue duration (5.2%), or for saccades in the wrong direction (0.4%). The number of excluded trials are shown in Table 1.

**Table 1:** Mean number of excluded trials for each condition.

	SOA 300 ms						SOA 1000 ms					
	Neutral			Fearful			Neutral			Fearful		
Excluded trials	Congruent	Incongruent	Direct gaze	Congruent	Incongruent	Direct gaze	Congruent	Incongruent	Direct gaze	Congruent	Incongruent	Direct gaze
anticipatory eye movements	0.5 (1.2)	0.0 (0.0)	0.0 (0.0)	0.8 (1.4)	0.0 (0.0)	0.1 (0.2)	0.3 (0.7)	0.1 (0.2)	0.3 (0.6)	0.4 (0.8)	0.2 (0.4)	0.4 (0.8)
exceeding the three standard deviation	0.4 (1.0)	0.5 (1.0)	0.6 (1.0)	0.4 (0.6)	0.7 (1.2)	0.5 (1.1)	0.3 (0.6)	0.4 (0.7)	0.6 (1.0)	0.5 (0.8)	0.6 (1.4)	0.7 (1.1)
blinks or poor validity	5.9 (6.3)	4.3 (5.3)	5.0 (5.1)	4.3 (5.5)	4.5 (5.8)	4.5 (5.7)	7.3 (8.0)	6.9 (7.5)	6.8 (6.2)	7.6 (7.6)	5.9 (5.3)	5.1 (6.9)
saccades in the wrong direction	0.0 (0.0)	0.8 (1.1)	0.1 (0.5)	0.0 (0.0)	1.1 (1.4)	0.2 (0.5)	0.0 (0.0)	0.3 (0.4)	0.0 (0.0)	0.0 (0.0)	0.3 (0.7)	0.0 (0.0)
the less than half of the face cue duration	2.7 (5.1)	1.9 (3.7)	2.3 (3.8)	1.7 (3.9)	2.1 (4.4)	2.3 (4.8)	3.2(5.5)	3.2 (5.3)	3.7 (6.1)	3.2 (5.4)	2.8 (3.9)	2.6 (5.0)

*Note*. Values in parentheses are standard deviations.

The ANOVA revealed that more trials were excluded in the neutral expression condition (M = 7.5, SD = 7.5) compared with the fearful expression condition (M = 6.9, SD = 6.9), *F* (1, 17) = 5.36, *MSE* = 19.56, *p* = .0333, η^2^_p_ = .24.

### Saccadic reaction times

First, the ANOVA revealed a significant main effect of gaze congruency, *F* (2, 34) = 7.22, *MSE* = 116.56, *p* = .002, η^2^_p_ = .30, with faster SRTs in the congruent condition (175.4 ms) than in the incongruent (181.3 ms) and the direct gaze condition (181.4 ms). A two-way interaction between SOA and gaze congruency was significant, *F* (2, 34) = 8.88, *MSE* = 55.7, *p* = .006, η^2^_p_ = .34, and a three-way interaction was also significant, *F* (2,34) = 4.57, *p* = .017, η^2^_p_ = .21. The other main effect and interaction were not statistically significant. Then to interpret the three-way interaction, we conducted separate two-way ANOVAs with facial expression and gaze congruency at each SOA condition.

**Fig 2:**
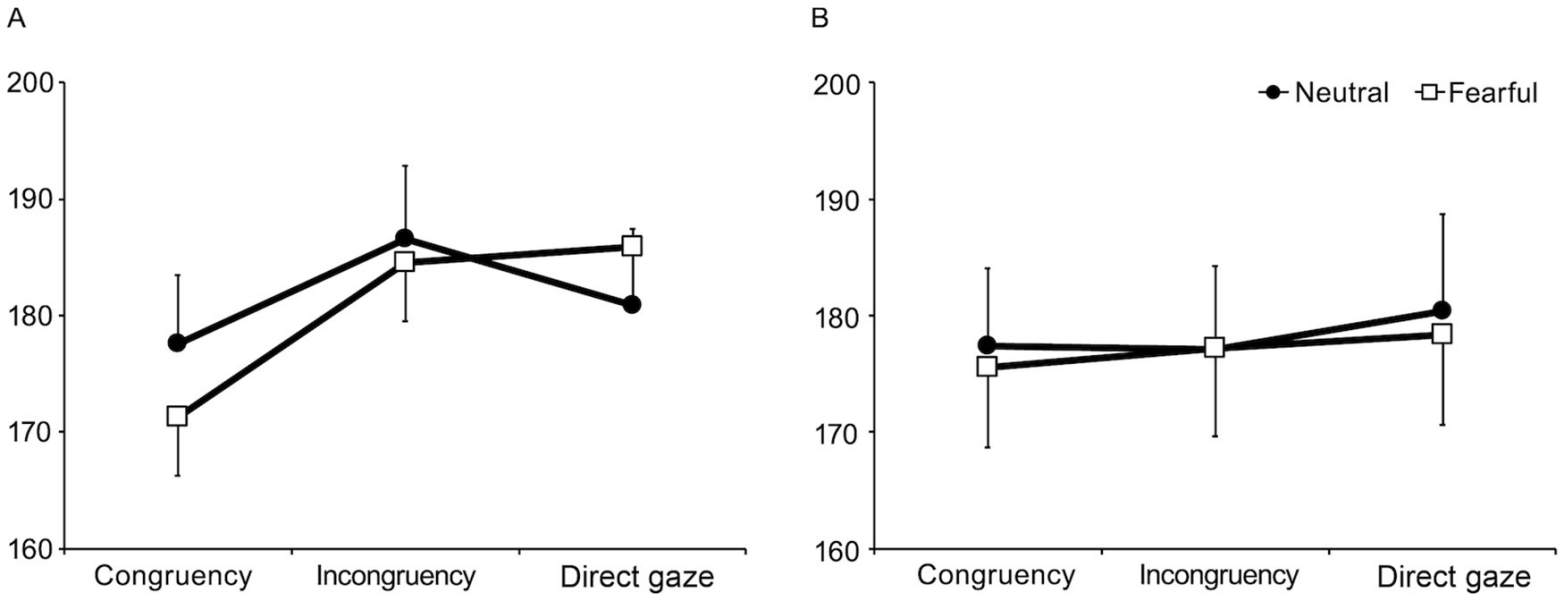
Mean saccadic reaction times (SRTs) for gaze congruency conditions among neutral and fearful expressions. (A) the 300 ms stimulus-onset-asynchrony (SOA) condition and (B) the 1000 ms SOA condition. Error bars indicate standard errors of the mean.

At the 300 ms SOA (Fig 2A), there was a significant main effect of gaze congruency, *F* (2, 34) = 11.89, *MSE* = 105.36, *p* < .001, η^2^_p_ = .41, with faster SRTs in the congruent condition (174.4 ms) than in the incongruent (185.5 ms) and the direct gaze condition (183.4 ms), *ps* < .0024. Importantly, the interaction between facial expression and gaze congruency was also significant, *F* (2, 34) = 5.88, *MSE* = 53.2, *p* = .006, η^2^_p_ = .30. First, as predicted, there were significant GCEs in both expressions; SRTs were shorter in the congruent condition than in the incongruent condition (*t* (17) = 2.97, *p* = .009 in the neutral expression; *t* (17) = 5.43, *p* < .001 in the fearful condition). The benefit of gaze cue was found, as SRTs were shorter in the congruent than in the direct gaze condition, in the fearful expression condition, *t* (17) = 5.21, *p* < .001. Second, the simple main effect of facial expression was significant under the congruent condition, *F* (1, 17) = 6.89, *MSE* = 52.55, *p* = .018, η^2^_p_ = .29, and marginally significant under the direct gaze condition, *F* (1, 17) = 3.74, *MSE* = 59.41, *p* = .070, η^2^_p_ = .18. In the congruent condition, SRTs were shorter in the fearful expression condition (171.3 ms) than in the neutral expression condition (177.6 ms). In the direct gaze condition, SRTs were numerically longer in the fearful expression condition (185.9 ms) than in the neutral expression condition (180.9 ms).

With respect to the magnitude of GCEs, planned t-test showed that the marginal significant effect of fearful expression was larger than that of neutral expression (9.0 ms and 13.3 ms in neutral expression and fearful expression respectively; *t* (17) = 3.58, *p* = .076).

At the 1000 ms SOA, there were no significant main effects nor interactions, *p*s > .20 (Fig 2B).

## Discussion

The present study investigated gaze-triggered attention orienting with neutral and fearful gaze by examining saccadic responses. The results revealed that: a) fearful averted gaze induced faster saccadic response toward the target, and b) both neutral and fearful gaze induced robust GCEs only at the short SOA. The findings indicate that eye movement behaviour is quite susceptible to fearful gaze, and also that information on gaze direction and facial expressions are rapidly integrated and influence our overt attention.

First, it is revealed that averted fearful gaze induced rapid saccadic response toward the gaze-cued target. At the neural level, it has been shown that the presence of fearful face facilitates early visual processing and improves visual detection threshold [31–33] and fearful gaze elicits the very early visual processing for the gaze-cued target [26]. The present result is in line with selective attentional effect with fearful gaze reported in neuroimaging research. Out of the previous studies using the manual responses, only a few reported the facilitated effect with fearful gaze on attention orienting [13, 14, 16]. There are a lot of variations in protocols; however, one possible factor is a presented cue sequence. Studies using a static cue sequence reported the null findings [8, 12], whereas studies using a dynamic cue sequence reported the emotional modulation (e.g. [11, 13–15]), especially a sequence in which the facial expression of emotion preceded, then followed, the gaze shift [14].

Another possible factor is the lower sensitivity of the manual response compared with the saccadic response to threat-related stimuli [20, 23]. Threat-related stimuli, such as fearful expression are rapidly processed in subcortical areas, especially in the amygdala [34, 35], and subcortical areas, such as brainstems and/or the superior colliculus (SC), play a major role in saccade generation [36]. The SC and the amygdala are supposed to have reciprocal projection [33, 37] or connections via pulvinar [38]; thus, it is possible that if fearful gaze is processed quickly in the amygdala, and the processed information is quickly conveyed to SC, then the effect of fearful gaze may be traceable in saccadic response. The facilitated saccadic response with fearful gaze was also found in infants [25], and it suggests that fearful gaze facilitates our saccadic response from the early stages of life.

Furthermore, RTs are commonly larger than SRTs; therefore, it is also possible that top-down modulation may occur in manual responses. With high-anxious people, it is reported that they have difficulty suppressing processing of threat-related distractors [39]; and this difficulty would result in the large magnitude of the effect with fearful gaze as reported in previous studies [7–9, 18]. On the other hand, recent research suggested that the magnitude of GCE with fearful gaze did not vary as a function of self-reported trait anxiety [14, 15, 40]. In the present study, we conducted the experiment among an unselected sample of people; therefore, further research is needed to clarify whether participant’s anxiety traits and/or autistic traits [17] relate to the magnitude of the GCE in the saccade response mode with fearful gaze.

Second, as we expected, the GCEs were found in both neutral and fearful expression conditions, and the magnitude of the effect was numerically larger for the fearful expression. These findings extend previous studies by showing that not only neutral gaze [5, 6] but also fearful gaze triggers overt attention orienting toward the gaze-cued side. With regard to the magnitude of the GCE, the marginal difference between neutral and fearful expression may be derived from the shorter SRTs with fearful expression in the congruent condition; the SRTs were not statistically different in the incongruent condition. The manner in which information of gaze direction and facial expression are represented in a saccade map [41, 42] remains unclear. The present findings suggest that information of gaze direction and facial expression are processed and integrated rapidly and represented in a saccade map.

Third, the effects of eye gaze and facial expressions were found only in the short SOA (300 ms) condition, and were not found in the long SOA (1000 ms) condition. Previous research using the saccade response mode reported similar results: the effect was found at the 500 ms SOA condition, but not at the 1000 ms SOA [5]. It has also been shown that the GCE was short-lived in the manual response mode [3–5]; however, if the SOA is set long enough (e.g. 2400 ms), the inhibition of return (IOR) would be observed [27], and it suggests that information of the gaze direction has been held and represented in an attentional map over 1000 ms. The lack of GCEs at the 1000 ms SOA condition in the present study might reflect not only a decay of facilitation but also an inhibitory effect by gaze cue. Additionally, a previous study using the manual response mode reported that the effect of fearful gaze emerged at the long SOA (more than 500 ms) condition [11]; however, no such effect was found in the present study. We did not directly compare saccadic and manual responses; but this finding is supported by previous studies, which have shown that the effect of threat-related stimuli is shorter lived for the saccade response mode than for the manual response mode [20, 22–24, 28, 43]. Recent research has shown that the oculomotor system is sensitive to threat-related stimuli (for a review, [23]), and that subcortical areas, such as the amygdala and/or the SC, would play an important role in rapid response toward a potential danger based on a limited amount of coarse visual information [33–35]. At the long SOA, the impact of rapid appraisal of averted gaze with fearful expression might diminish and the facilitated effect by fearful gaze might disappear on the saccadic response. Further research is needed to investigate the time course of fearful averted gaze effects on overt attention orienting.

In conclusion, the present study shows that fearful averted gaze induced more rapid saccadic response than a neutral one at the short SOA (300 ms), and the magnitudes of the effects were numerically larger for a fearful expression than for a neutral one. The rapid saccadic response toward a fearful gaze-cued target is in line with previous neuroimaging findings that fearful gaze cue elicits selectively early target processing. By examining saccadic response, we successfully captured the effect of fearful gaze on our attention orienting. Future research is needed to examine whether this facilitated saccadic response is limited to fearful averted gaze, and whether the facilitated saccadic response varies as a function of participant traits. The manner in which social signals influence the oculomotor system is an engaging topic for future research.
